# Predictors of mosaic chromosome Y loss and associations with mortality in the UK Biobank

**DOI:** 10.1038/s41598-018-30759-1

**Published:** 2018-08-17

**Authors:** Erikka Loftfield, Weiyin Zhou, Barry I. Graubard, Meredith Yeager, Stephen J. Chanock, Neal D. Freedman, Mitchell J. Machiela

**Affiliations:** 1 Division of Cancer Epidemiology and Genetics, National Cancer Institute, Bethesda, Maryland USA; 2 Cancer Genomics Research Laboratory, Frederick National Laboratory for Cancer Research, Leidos Biomedical Research, Inc., Frederick, Maryland USA

## Abstract

Mosaic loss of the Y chromosome (mLOY) is the most commonly reported large structural somatic event. Previous studies have indicated age and cigarette smoking increase the risk of mLOY, but the relationship of other exposures with mLOY and mLOY with disease has not been adequately investigated. We characterized mLOY in a large cohort of 223,338 men from the UK Biobank by scanning for deviations in genotyping array median log_2_ intensity ratios (mLRR) of the Y chromosome using a standard algorithm. A total of 3,789 (1.7%) men showed evidence for mLOY (mLRR < −0.15). In multivariable-adjusted logistic regression models, we found that mLOY increases exponentially with age (overall P-value < 4.9 × 10^−324^; p-value for the quadratic term = 2.1 × 10^−7^), and observed a strong association with current smoking (P-value = 7.8 × 10^−184^). We observed less mLOY in men of African ancestry (0.4%) compared to men of European ancestry (1.8%, P-value = 0.003). Although mLOY was not associated with prevalent cancer (P-value = 0.61), associations were observed for diabetes (P-value = 0.003) and cardiovascular disease (P-value = 0.01). Using Cox proportional hazards regression models, mLOY was associated with all-cause mortality among men with a high proportion of cells affected (mLRR < −0.40; HR = 1.35, 95% CI = 1.08–1.70, P-value = 0.009). In conclusion, mLOY was associated with several health-related factors as well as with all-cause mortality. Further functional studies are warranted to understand how and in what way mLOY could influence adult male health.

## Introduction

Mosaicism is the acquisition of post-zygotic mutations in a clonal sub-population of cells^1,2^. Large epidemiologic studies have reported mosaic mutations in blood, buccal epithelial cells, skin and neurons^3–10^, with mutations ranging in size from mosaic single nucleotide variants (SNV) to large structural events that encompasses entire chromosomes (e.g., a chromosomal gain or deletion)^8,11,12^ Large surveys have indicated that mosaicism of the sex chromosomes is more common than autosomal structural events^4,5,8,13–15^, of which the most frequently observed large structural mosaic event is loss of the Y chromosome^13,15^. Mosaic loss of the Y chromosome (mLOY) increases in frequency with age, perhaps reaching a frequency in the range of 15–20% in men over 80 years of age^15^.

Recent studies have identified additional risk factors associated with acquiring mLOY. After age, the strongest association is cigarette smoking^15,16^. Current smokers are at higher risk of mLOY than former smokers, and former smokers remain at elevated risk for up to 20 years post-cessation relative to never smokers^15^. Genetic susceptibility loci have also been identified. An initial genome-wide association study identified a locus near *TCL1A* as a risk factor for mLOY^15^. A subsequent, larger study in the initial release of the UK Biobank replicated this locus and discovered 18 additional loci^17^, providing further evidence for a genetic contribution to the development of mLOY. Previous studies also found evidence suggesting mLOY may be associated with both disease incidence and survival^13,15,18–20^, although associations with cancer incidence and survival have been inconsistent due to issues of small sample size and study design^13,15,18^. Recent studies have also observed preliminary evidence suggesting associations between mLOY in blood and cardiovascular disease, Alzheimer’s disease and suicide^19–21^.

Herein, we performed a study of 223,338 men from the UK Biobank, a large UK study with available genetic data and both extensive exposure and outcome characterization^22^. We scanned intensity data from the Y chromosome using both a continuous measure (mLRR) and two dichotomous measures (mLRR < −0.15 and mLRR < −0.40) to identify evidence for mLOY in a detectible percentage of circulating blood cells to better understand the determinants and distribution of mLOY in UK men.

## Methods

### Study design and participants

The design of the UK Biobank has been described in detail elsewhere^22,23^. In brief, invitations were mailed to approximately 9.2 million individuals in the UK’s National Health Service, aged 40 to 69 years, who resided within 40 kilometers of 22 assessment centers across the UK^22^. In total, 503,317 individuals visited an assessment center between 2006 and 2010^23^ and provided baseline information on demographic, lifestyle and other health-related factors, biological samples, and physical measures. Genetic data from 223,507 male participants was made available by the UK Biobank for our study. We further excluded participants with sex discrepancies between the self-reported and inferred sex using X-chromosome heterozygosity (n = 167) as well as those with no follow-up time (n = 2), resulting in a final analytic cohort of N = 223,338 participants.

The UK Biobank study was approved by the National Information Governance Board for Health and Social Care and the National Health Service North West Multicentre Research Ethics Committee^22,23^. All participants provided signed informed consent at enrollment and all research was performed in accordance with relevant guidelines/regulations. All data used in this analysis is available through application to the UK Biobank.

### Cohort follow-up

Follow-up time was counted from the date of assessment center visit, at which time blood was drawn and baseline information ascertained, until the date of death or the date of censor (i.e., January 31, 2016 for England and Wales and November 30, 2015 for Scotland), whichever came first. For cause-specific mortality analyses, individuals who died from other causes were censored at their date of death.

### Ascertainment of death

Vital status, date, and primary cause of death were provided by the National Health Service (NHS) Information Centre for participants from England and Wales and by the NHS Central Register, Scotland for those from Scotland. For cause-specific mortality we used the International Classification of Diseases, edition 10 (ICD-10) codes to define all-cancer (C00-D48) and all-cardiovascular disease (I00-I79) mortality. In addition, we further defined common causes of death (i.e., causes with >250 deaths) within these broad categories as follows: digestive system cancer (C15-C26 and C48), respiratory system cancer (C30-C39), ischemic heart diseases (I20-I25), and stroke (I60-I69).

### Assessment of exposures

During the UK Biobank Assessment Centre visit, participants completed a touchscreen questionnaire that queried demographic, lifestyle, and other health-related factors. From this self-reported data, we created a 25-level detailed smoking history variable by combining data on smoking status, lifetime smoking, smoking intensity, time since quitting for former smokers, and type of tobacco smoked. We created a 6-level variable for alcohol drinking by combining data on drinking status and amount of alcohol consumed per week, calculated as the sum of all alcoholic beverages consumed on average per day, and we created a 4-level variable for physical activity by combining data on frequency of moderate or vigorous activity. Categories of body mass index (BMI), were defined according to the definition of the World Health Organization^24^. Prior diagnoses of diabetes, cancer (other than non-melanoma skin cancer), and heart attack or stroke were obtained via self-report. For diabetes, the self-report questionnaire did not distinguish between type 1 and type 2 diabetes; however, fewer than 2% of cases were diagnosed earlier than age 44 suggesting the majority of cases were type 2 diabetes. Indicator variables were used to account for missing data in regression models.

UK Biobank participants were genotyped using genome-wide arrays. The initial 50,000 participants were genotyped using the Affymetrix UK BiLEVE Axiom array, and the remaining were genotyped using the Affymetrix UK Biobank Axiom® array. Both arrays had 691 markers across male specific region of chromosome Y (MSY) (chrY:2658271-28767492, hg19/GRCh37). Quality control (QC) was performed centrally by the Wellcome Trust Centre for Human Genetics as described elsewhere^25^. Each subject has a reported Log_2_ Intensity Ratio (LRR) and B-Allele Frequency (BAF) available. Y chromosome mosaicism was detected using LRR, which is the normalized measure of total signal intensity and provides data on relative copy number. Subjects were examined for deviations from expected median LRR (mLRR) for evidence of loss of the male specific region of chromosome Y (MSY). Evidence for loss is reflected by negative mLRR values, while evidence for gain is reflected by positive mLRR values. We defined mLOY dichotomously using two-different cut-points that have been previously cited in the literature: mLRR <−0.15 and mLRR <−0.40^13,15^. These cutpoints represent cellular proportions of approximately 10% (1–2^−0.15^) and 24% (1–2^−0.40^), although molecular confirmation was not possible in the UK Biobank. A mLRR >+0.15 was used to define a Y chromosome gain event. For potential mLOY, each chromosome Y plot was then manually reviewed and suspect events were further excluded from subsequent analyses. For subjects with very low mLRR (<−0.95), their chromosome X plots were manually examined to confirm they are indeed males. We also performed analyses using continuous mLRR. For ease of interpretation we scaled the continuous mLRR variable by the standard deviation of the mLRR divided by −1 such that risk estimates are interpreted as a one standard deviation decrease in the mLRR. Frequency plots were generated in R using the binom package.

### Statistical analysis

We tabulated our two definitions of mLOY and mLRR by demographic, lifestyle, and other health-related factors and used multivariable-logistic regression models to test for an association between each factor of interest and mLOY or mLRR, adjusting for continuous age and age-squared and the other variables in Table 1. We used multivariable Cox proportional hazard regression models to estimate hazard ratios (HR) and 95% confidence intervals (CI) for all-cause and cause-specific mortality. Age was used as the underlying time metric since we expected the hazard to change more as a function of age than as a function of time enrolled in the study^26^. We tested the proportional-hazards assumption by comparing the multivariable model with the interaction term between person-time and mLRR to the model without it using the likelihood ratio test. Detecting a deviation from this assumption for mLRR and all-cause mortality (P-value for likelihood ratio test = 0.005), we evaluated associations in the first and second halves of follow-up to better understand how HR estimates changed over time.

**Table 1:** Baseline characteristics and mosaic loss of the Y chromosome.

	Entire Cohort	mLOY (mLRR <−0.15)		mLOY (mLRR <−0.40)		mLRR	
	N (%)	N (%)	Adj. P-value^a^	N (%)	Adj. P-value^a^	median ± SD	Adj. P-value^a^
**Age**							
<65 years	187,711 (84.0)	1,901 (50.2)	ref	260 (43.6)	ref	0.007 ± 0.052	ref
≥65 years	35,627 (16.0)	1,888 (49.8)	<4.9 × 10^−324^	336 (56.4)	4.6 × 10^−99^	−0.008 ± 0.085	<4.9 × 10^−324^
**Smoking status**							
Never smoker	108,859 (49.0)	1,012 (26.9)	ref	126 (21.2)	ref	0.007 ± 0.051	ref
Former smoker	85,537 (38.5)	1,808 (48.0)	2.8 × 10^−24^	282 (47.5)	2.8 × 10^−8^	0.003 ± 0.063	1.2 × 10^−18^
Current smoker	27,748 (12.5)	946 (25.1)	7.8 × 10^−184^	186 (31.3)	7.9 × 10^−50^	0.001 ± 0.076	1.0 × 10^−281^
**Race/Ethnicity**							
White	210,179 (94.6)	3,679 (97.7)	ref	586 (98.5)	ref	0.004 ± 0.060	ref
Mixed Race	1,070 (0.5)	5 (0.1)	0.06	1 (0.2)	0.51	0.009 ± 0.051	0.36
Asian	5,671 (2.6)	49 (1.3)	0.13	6 (1)	0.26	0.010 ± 0.050	0.001
Black	3,285 (1.5)	12 (0.3)	0.003	0 (0)	0.96	0.017 ± 0.044	1.6 × 10^−16^
Other	1,882 (0.8)	19 (0.5)	0.77	2 (0.3)	0.62	0.012 ± 0.048	0.01
**Self-reported health status**							
Excellent	34,777 (15.7)	480 (12.8)	ref	68 (11.5)	ref	0.005 ± 0.055	ref
Good	124,573 (56.1)	2,074 (55.1)	0.30	316 (53.3)	0.49	0.005 ± 0.058	0.58
Fair	51,063 (23)	971 (25.8)	0.03	166 (28)	0.08	0.005 ± 0.062	0.91
Poor	11,527 (5.2)	239 (6.4)	0.009	43 (7.3)	0.06	0.004 ± 0.069	0.07
**Body mass index**							
Underweight (<18.5 kg/m^2^)	483 (0.2)	7 (0.2)	0.25	1 (0.2)	0.46	0.005 ± 0.055	0.38
Normal (18.5 to <25 kg/m^2^)	53,505 (24.4)	914 (24.7)	ref	151 (25.9)	ref	0.004 ± 0.060	ref
Overweight (25 to <30 kg/m^2^)	108,768 (49.7)	1,942 (52.5)	0.50	310 (53.1)	0.57	0.004 ± 0.060	0.003
Obese I (30 to <35 kg/m^2^)	43,676 (19.9)	675 (18.3)	0.0001	98 (16.8)	0.01	0.005 ± 0.058	2.7 × 10^−12^
Obese II/III (≥35 kg/m^2^)	12,609 (5.8)	159 (4.3)	0.0006	24 (4.1)	0.07	0.008 ± 0.057	6.7 × 10^−17^
**Physical activity (>10 minutes of moderate or vigorous activity)**							
0 days/week	23,637 (11.4)	430 (12.7)	0.21	79 (15.0)	0.08	0.004 ± 0.061	0.14
1 or 2 days/week	28,237 (13.6)	413 (12.2)	0.75	57 (10.8)	0.46	0.005 ± 0.056	0.16
2 or 3 days/week	35,005 (16.8)	569 (16.8)	0.91	86 (16.3)	0.91	0.005 ± 0.058	0.35
≥5 days/week	121,165 (58.2)	2,002 (59.1)	ref	304 (57.8)	ref	0.005 ± 0.059	ref
**Alcohol drinking status**							
Never drinker	6,243 (2.8)	78 (2.1)	0.40	11 (1.8)	0.69	0.008 ± 0.055	0.14
Former drinker	7,883 (3.5)	144 (3.8)	0.56	23 (3.9)	0.70	0.003 ± 0.062	0.50
Current drinker (<1 drink/week)	36,187 (16.2)	553 (14.6)	0.37	88 (14.8)	0.82	0.006 ± 0.059	0.51
Current drinker (≥1 drink/week >7)	44,922 (20.2)	686 (18.2)	0.38	111 (18.6)	0.74	0.005 ± 0.058	0.66
Current drinker (1 to 3 drinks/day)	95,935 (43.1)	1,621 (42.9)	ref	249 (41.8)	ref	0.004 ± 0.059	ref
Current drinker (>3 drinks/day)	31,550 (14.2)	698 (18.5)	0.0005	114 (19.1)	0.13	0.004 ± 0.065	0.27
College or university degree	75,324 (41.4)	953 (36.3)	ref	133 (32.8)	ref	0.006 ± 0.054	ref
**Education level**							
A levels/AS levels or equivalent	22,915 (12.6)	322 (12.3)	0.28	60 (14.8)	0.04	0.006 ± 0.058	0.22
O levels/GCSEs or equivalent	41,341 (22.7)	677 (25.8)	0.25	100 (24.7)	0.78	0.005 ± 0.057	0.53
CSEs or equivalent	12,048 (6.6)	79 (3.0)	0.91	14 (3.5)	0.46	0.009 ± 0.050	0.68
NVQ or HND or HNC or equivalent	20,204 (11.1)	384 (14.6)	0.55	64 (15.8)	0.34	0.004 ± 0.061	0.2
Other qualifications	9,992 (5.5)	213 (8.1)	0.73	34 (8.4)	0.87	0.002 ± 0.064	0.58
**Diabetes diagnosis**							
No	206,681 (93.0)	3,451 (91.5)	ref	545 (91.6)	ref	0.005 ± 0.059	ref
Yes	15,507 (7.0)	319 (8.5)	0.003	50 (8.4)	0.14	0.004 ± 0.065	5.6 × 10^−13^
**Cancer (other than non-melanoma skin cancer) diagnosis**							
No	211,208 (94.6)	3,463 (91.4)	ref	541 (90.8)	ref	0.005 ± 0.059	ref
Yes	12,130 (5.4)	326 (8.6)	0.61	55 (9.2)	0.94	0.001 ± 0.069	0.14
**Heart attack or stroke diagnosis**							
No	211,710 (94.8)	3,399 (89.7)	ref	530 (88.9)	ref	0.005 ± 0.058	ref
Yes	11,628 (5.2)	390 (10.3)	0.01	66 (11.1)	0.32	−0.002 ± 0.075	1.4 × 10^−10^

^a^Adjusted P-values (Adj. P-value) from multivariable logistic regression models are adjusted for all other covariates in Table 1. In this multivariable adjusted model, we fit parameters for continuous age and age-squared. Abbreviations: mLOY, mosaic loss of the Y chromosome; mLRR, median log_2_ intensity ratio of the Y chromosome.

To explore the shape of the association between mLOY and mortality, we used a restricted cubic spline approach where the reference value for mLRR was set at zero for HR estimates with 5 knots set at the 1^st^, 25^th^, 50^th^, 75^th^ and 95^th^ percentiles of mLRR. To test for a potential nonlinear association between mLOY and mortality risk, we compared the model with only the linear term for mLOY with the model containing both the linear and the cubic spline terms using a likelihood ratio test. For the spline analysis, we restricted the analytic sample to those without a Y chromosome gain event since only 11 deaths occurred among this small population subset.

In secondary analyses, we ran multivariable-adjusted Cox regression models stratified by the following potential effect modifiers: age, smoking status, and general health status. We assessed potential effect modification by modeling the cross-product term of the stratifying variable with mLRR; the P-value for interaction corresponds to the likelihood ratio test comparing the multivariable models with and without the cross-product terms for each level of the stratifying variable. To better understand the potential impact of prevalent disease on the association, we conducted sensitivity analyses excluding individuals with a self-reported chronic disease (i.e., cancer, diabetes, heart attack or stroke) at baseline. Finally, we conducted a sensitivity analysis excluding 205 outliers with a potential mosaic chromosome Y gain (mLRR >+0.15). All statistical tests were two-sided and P-values of < 0.05 without correction for multiple comparisons were interpreted as statistically significant. We used SAS software version 9.4 (SAS Institute, Cary, North Carolina) and the computational resources of the NIH’s High-Performance Computing Biowulf cluster to conduct analyses.

### Role of funding source

This study was an investigator-initiated project and was supported by the Intramural Research Program of the National Institutes of Health, Division of Cancer Epidemiology and Genetics, National Cancer Institute, Department of Health and Human Services. The study sponsor had no role in the design of the study, data collection, data analysis, data interpretation, or writing of the manuscript. The corresponding author had full access to all the data in the study and final responsibility for the decision to submit for publication.

## Results

The hybridization data from all males were examined for deviations from expected mLRR (Methods, Supplementary Fig. 1) for evidence of loss or gain of the male specific region of chromosome Y. Both a continuous measure of chromosome Y loss (i.e., mLRR) and dichotomous indicators of Y loss (i.e., mLRR <−0.15 and mLRR <−0.40) were created. Among the 223,338 males aged 37–73 (mean = 57, median = 58) in final analytic cohort, a total of 3,789 men (mLRR <−0.15, 1.7%) had detectable mLOY and of these men 596 (16%) had high proportions of cells affected (mLRR <−0.40). We also found evidence of chromosome Y gain for 205 males (mLRR > 0.15, 0.09%).

**Figure 1:**
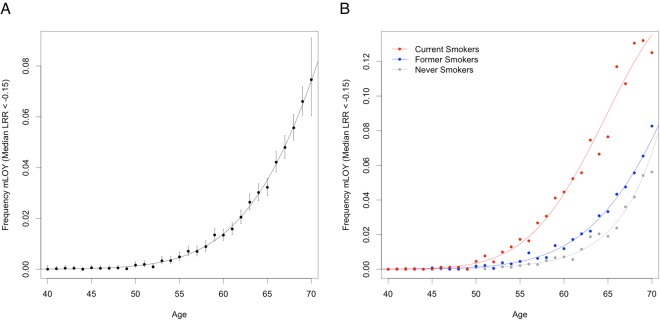
Frequency of mLOY (mLRR <−0.15) by age of blood draw in (**A**) our analytic set of all males in the UK Biobank and (**B**) by smoking status. Points represent the estimated frequency of mLOY for each age. Error bars in (**A**) are 95% confidence intervals around the frequency point estimates. Plotted curves are estimated from logistic regression models of mLOY with age and age^2^ as the predictor variables. Abbreviations: mLOY, mosaic loss of the Y chromosome; mLRR, median log_2_ intensity ratio of the Y chromosome.

As previously reported, we observed a robust association between age and frequency of mLOY (mLRR P-value < 4.9 × 10^−324^, Table 1). We note that the overall proportion of men with mLRR <=−0.15 is negligible before 50 years of age and then rapidly increases with age (Fig. 1A). Supporting this observation, we observed evidence for an exponential increase in mLOY with age such that a model including both age and age-squared (P-value = 2.1 × 10^−7^) fit the association better than a model with age alone.

A higher proportion of current smokers (3.4%) were affected by mLOY (mLRR <−0.15) than former smokers (2.1%) and non-smokers (0.9%). Marked differences by smoking status were consistent across increasing age (Fig. 1B) (Table 1). Novel associations with self-reported ancestry, health, and body mass index (BMI) were observed with mLOY (mLRR <−0.15, Table 1). A lower percentage of black (0.4%) as compared with white (1.8%) men were affected by mLOY (P-value = 0.003). Men reporting poor health had a higher proportion of mLOY than men reporting excellent health (2.1% vs 1.4%, P-value = 0.009). For BMI, a higher proportion of mLOY was observed among men with a BMI in the normal range relative to men classified as obese class I (1.7% vs. 1.5%, P-value = 0.0001) or obese class II/III (1.7% vs. and 1.3%, P-value = 0.0006). We also observed higher proportions of mLOY among men with a prior diagnosis of diabetes (2.6% vs. 1.6%, P-value = 0.003), heart attack, or stroke (3.4% vs. 1.6%, P-value = 0.01). Similar associations were observed when using a more extreme mLOY threshold (mLRR <−0.40) or mLRR; however, the statistical significance of observed associations varied considerably (Table 1).

**Figure 2:**
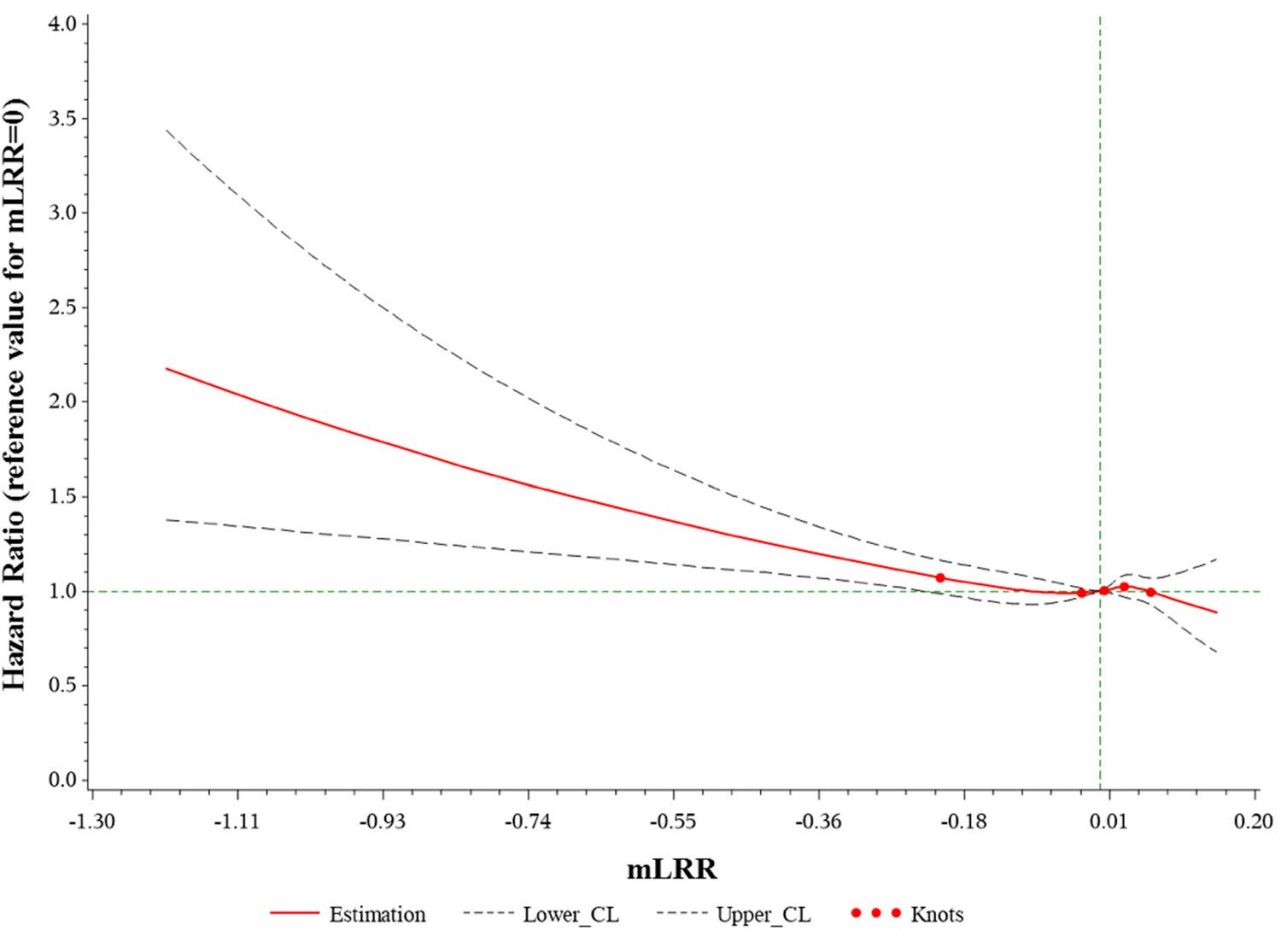
Multivariable-adjusted Cox proportional hazards regression for mLRR and all-cause mortality, modeled with a restricted cubic spline with knots at the 1^st^, 25^th^, 50^th^, 75^th^, and 95^th^ percentiles of mLRR, illustrating the relationship between mosaic loss of the Y chromosome and all-cause mortality (P-value of overall association = 0.01; P-value of nonlinear association = 0.12). Abbreviations: mLRR, median log_2_ intensity ratio of the Y chromosome.

Over 10 years of follow-up (median 7 years) and 1.5 million person-years, 8,401 deaths occurred. In our primary analysis of mLOY with all-cause mortality (Table 2), we observed an association with mLOY with a higher (mLRR <−0.40, HR = 1.35, 95% CI = 1.08–1.70, P-value = 0.009), but not a lower proportion of cells with mLOY (mLRR <−0.15, HR = 1.08, 95% CI = 0.97–1.21, P-value = 0.16). We also observed suggestive evidence of a linear association with all-cause mortality (HR = 1.02, 95% CI = 1.00–1.03, P-value = 0.07) using the continuous variable mLRR. These findings were supported by a spline analysis which visually presents the association between mLRR and mortality (Fig. 2). No association with mortality was observed for mosaic gain of the Y chromosome in the small numbers available. For cause-specific mortality (Table 3), we found a higher risk of cancer death among men with a higher proportion of affected cells (mLRR <−0.40, HR = 1.48, 95% CI = 1.10–1.99, P-value = 0.01). We did not observe an association between mLOY and cardiovascular disease mortality, although the number of deaths among men with mLOY was small (mLRR <−0.15 = 77 deaths, mLRR <−0.40 = 12 deaths).

**Table 2:** Hazard ratios and 95% confidence intervals for mosaic loss of the Y chromosome (mLOY) and all-cause mortality using age as the underlying time metric (N = 223, 338).

	mLOY (mLRR <−0.15)			mLOY (mLRR <−0.40)			mLRR^d^		
	HR	95% CI	P-value	HR	95% CI	P-value	HR	95% CI	P-value
N (%) with mLOY	3,789 (1.70)			596 (0.27)					
No. deaths	8401			8401			8401		
No. deaths with mLOY	334			75					
Age-adjusted^a^	1.37	(1.23–1.53)	1.7 × 10^−8^	1.88	(1.50–2.36)	6.0 × 10^−8^	1.06	(1.04–1.08)	9.0 × 10^−12^
Age- & smoking-adjusted^b^	1.06	(0.95–1.18)	0.31	1.35	(1.08–1.70)	0.01	1.01	(0.99–1.03)	0.24
Multivariable-adjusted^c^	1.08	(0.97–1.21)	0.16	1.35	(1.08–1.70)	0.009	1.02	(1.00–1.03)	0.07

^a^Adjusted for age using age as the underlying time metric in the Cox proportional hazards regression model.

^b^Additionally adjusted for detailed smoking history (25-level variable incorporating current smoking status, smoking intensity (current and former smokers); time since quitting (former smokers), and cigar and pipe use (current and former smokers).

^c^Additionally adjusted for race/ethnicity (white, black, Asian, mixed, or other race); alcohol drinking (never drinker, former drinker, infrequent drinker (<1 drink/week), occasional drinker (>1 drink/week but <1 drink/day), moderate daily drinker (1 to 3 drinks/day), or heavy daily drinker (>3 drinks/day)); general health status (excellent, good, fair, or poor); education level (college or university degree, A levels/AS levels or equivalent, O levels/GCSEs or equivalent, CSEs or equivalent, NVQ or HND or HNC equivalent, or other professional qualifications); body mass index (<18.5, 18.5 to <25, 25 to <30, 30 to <35, or ≥35 kg/m^2^); and physical activity (>10 minutes of moderate of vigorous activity 0, 1-2, 3-4, or ≥5 days/week).

^d^Scaled by the - (standard deviation) of mLRR such that the HR corresponds to a one standard deviation decrease in mLRR. Abbreviations: CI, confidence interval; HR, hazard ratio; mLOY, mosaic loss of the Y chromosome; mLRR, median log_2_ intensity ratio of the Y chromosome.

**Table 3:** Hazard ratios and 95% confidence intervals for mosaic loss of the Y chromosome and cause-specific mortality using age as the underlying time metric (N = 223, 338).

	mLOY (mLRR <−0.15)			mLOY (mLRR <−0.40)			mLRR^b^		
	HR^a^	95% CI	P-value	HR^a^	95% CI	P-value	HR^a^	95% CI	P-value
Cancer mortality	1.04	(0.89-1.21)	0.62	1.48	(1.10-1.99)	0.01	1.02	(0.99-1.04)	0.19
No. deaths with mLOY/total deaths	174/4397			44/4397					
Respiratory system cancer mortality	1.03	(0.77-1.39)	0.87	1.65	(0.98-2.75)	0.06	1.05	(1.01-1.10)	0.01
No. deaths with mLOY/total deaths	48/840			15/840					
Digestive system cancer mortality	0.88	(0.65-1.19)	0.4	1.19	(0.64-2.23)	0.58	0.98	(0.94-1.02)	0.35
No. deaths with mLOY/total deaths	43/1464			10/1464					
Cardiovascular disease mortality	1.05	(0.83-1.32)	0.70	0.93	(0.52-1.64)	0.79	1.00	(0.97-1.04)	0.91
No. deaths with mLOY/total deaths	77/2069			12/2069					
Ischemic heart disease mortality	1.05	(0.78-1.40)	0.77	1.13	(0.59-2.19)	0.71	1.01	(0.97-1.06)	0.58
No. deaths with mLOY/total deaths	48/1322			9/1322					
Stroke mortality	1.56	(0.94-2.57)	0.08	0.96	(0.24-3.90)	0.96	1.02	(0.94-1.11)	0.67
No. deaths with mLOY/total deaths	17/293			2/293					

^a^Multivariable model is adjusted for age (as the underlying time metric), detailed smoking history (25-level variable incorporating current smoking status, smoking intensity (current and former smokers); time since quitting (former smokers), and cigar and pipe use (current and former smokers)); time to first cigarette among current smokers (<5 minutes, 5 to 15 minutes, 30 minutes to 1 hour, or >1 hour)); race/ethnicity (white, black, Asian, mixed, or other race); alcohol drinking (never drinker, former drinker, infrequent drinker (<1 drink/week), occasional drinker (>1 drink/week but <1 drink/day), moderate daily drinker (1 to 3 drinks/day), or heavy daily drinker (>3 drinks/day); general health status (excellent, good, fair, or poor); education level (college or university degree, A levels/AS levels or equivalent, O levels/GCSEs or equivalent, CSEs or equivalent, NVQ or HND or HNC equivalent, or other professional qualifications); body mass index (<18.5, 18.5 to <25, 25 to <30, 30 to <35, or ≥35 kg/m^2^); and physical activity (>10 minutes of moderate of vigorous activity 0, 1-2, 3-4, or ≥5 days/week).

^b^Scaled by the - (standard deviation) of mLRR such that the HR corresponds to a one standard deviation decrease in mLRR. Abbreviations: CI, confidence interval; HR, hazard ratio; mLOY, mosaic loss of the Y chromosome; mLRR, median log_2_ intensity ratio of the Y chromosome.

In secondary analyses, HR estimates for mLOY and all-cause mortality did not differ by self-reported health status (P-value for interaction = 0.79). Associations with mortality were noted only in ever-smokers and were stronger among younger participants, although differences by age (P-value for interaction = 0.38) and smoking status (P-value for interaction = 0.46) were not statistically significant (Table 4). Excluding individuals with a Y chromosome gain event did not markedly alter HR estimates (Supplementary Table 1). In a sensitivity analysis, HR estimates were attenuated for the −0.15 mLOY threshold when those with a prior diagnosis of diabetes, cancer, heart attack or stroke were excluded from the analysis (HR = 1.06, 95% CI = 0.91–1.22, P-value = 0.47) (Supplementary Table 2). Finally, the lag analysis indicated that baseline mLRR <−0.15 was associated with deaths occurring in years 5 to 10 of follow-up (HR = 1.26, 95% CI = 1.06–1.49, P-value = 0.008), but not in the first five years of follow-up, (HR = 1.00, 95% CI = 0.86–1.16, P-value = 0.98) (Supplementary Table 3). In contrast, associations with a mLRR <−0.40 were similar in magnitude and direction among deaths occurring in the first five and later five years of follow-up, although due to small numbers was not statistically significant in the later five years of follow-up.

**Table 4:** Hazard ratios and 95% confidence intervals for mosaic loss of the Y chromosome and all-cause mortality using age as the underlying time metric and stratified by baseline characteristics.

	mLOY (mLRR <−0.15)			mLOY (mLRR <−0.40)			mLRR ^d^		
	HR	95% CI	P-value	HR	95% CI	P-value	HR	95% CI	P-value
**Age** ^**a**^									
<65 years	1.15	(0.97–1.37)	0.10	1.43	(0.96–2.12)	0.08	1.01	(0.99–1.04)	0.43
≥65 years	1.05	(0.91–1.22)	0.51	1.35	(1.02–1.79)	0.04	1.02	(1.00–1.05)	0.04
**Smoking status** ^**b**^									
Never smoker	0.90	(0.67–1.22)	0.50	1.10	(0.52–2.32)	0.80	1.00	(0.96–1.04)	0.91
Former smoker	1.20	(1.02–1.40)	0.03	1.43	(1.02–2.02)	0.04	1.03	(1.00–1.05)	0.03
Current smoker	1.19	(1.00–1.43)	0.06	1.51	(1.08–2.11)	0.02	1.03	(1.01–1.06)	0.02
**Self-reported health** ^**c**^									
Excellent/Good	1.08	(0.92–1.26)	0.35	1.34	(0.96–1.87)	0.09	1.01	(0.99–1.04)	0.35
Fair/Poor	1.06	(0.91–1.24)	0.47	1.36	(0.99–1.86)	0.06	1.02	(0.99–1.04)	0.17

^a^Multivariable model is adjusted for age (as the underlying time metric), detailed smoking history (25-level variable incorporating current smoking status, smoking intensity (current and former smokers); time since quitting (former smokers), and cigar and pipe use (current and former smokers)); time to first cigarette among current smokers (<5 minutes, 5 to 15 minutes, 30 minutes to 1 hour, or >1 hour)); race/ethnicity (white, black, Asian, mixed, or other race); alcohol drinking (never drinker, former drinker, infrequent drinker (<1 drink/week), occasional drinker (>1 drink/week but <1 drink/day), moderate daily drinker (1 to 3 drinks/day), or heavy daily drinker (>3 drinks/day); general health status (excellent, good, fair, or poor); education level (college or university degree, A levels/AS levels or equivalent, O levels/GCSEs or equivalent, CSEs or equivalent, NVQ or HND or HNC equivalent, or other professional qualifications); body mass index (<18.5, 18.5 to <25, 25 to <30, 30 to <35, or ≥35 kg/m^2^); and physical activity (>10 minutes of moderate of vigorous activity 0, 1-2, 3-4, or ≥5 days/week).

^b^Smoking stratified models were not adjusted for detailed smoking history.

^c^Self-reported health stratified models were not adjusted for self-reported health.

^d^Scaled by the - (standard deviation) of mLRR such that the HR corresponds to a one standard deviation decrease in mLRR. Abbreviations: CI, confidence interval; HR, hazard ratio; mLOY, mosaic loss of the Y chromosome; mLRR, median log_2_ intensity ratio of the Y chromosome.

## Discussion

We investigated mLOY in a large cohort of 223,338 men from the UK Biobank by scanning for deviations in mLRR of the Y-specific region of the male Y chromosome. We analyzed mLOY at mLRR <−0.15 and mLRR <−0.40 cutpoints as well as continuous mLRR. Our study is the largest to date, providing sufficiently strong statistical power to replicate prior cross-sectional associations for mLOY with age and smoking, as well as uncover new associations with ancestry, self-reported health, BMI, self-reported diabetes, and self-reported heart attack or stroke. Furthermore, men with a high proportion of cells with mLOY had a higher risk of mortality during follow-up.

The UK Biobank provides a unique opportunity to examine both the predictors and consequences of mLOY. Aging is associated with an accumulation of somatic mutations, so it is not surprising that rates of mLOY increase with age. Age-related mLOY has been previously reported^13,15^, but existing datasets were smaller and had limited ability to test for non-linear associations. In this analysis, we report evidence that the frequency of mLOY increases exponentially with age (i.e., a quadratic rather than a linear relationship). The prevalence of mLOY appears to remain low until approximately 50 years of age after which the prevalence rapidly increases. Accelerated stochastic processes coupled with inherited variation in genome maintenance^15,17^ likely influence risk of mLOY. In combination with these mechanisms, it is possible that age-related changes in stem cell compartment diversity and reduced levels of immuno-surveillance permit sub-populations of cells with mLOY to increase in abundance. Previous studies of autosomal mosaicism support this possibility, suggesting the proportion of cells affected by mosaicism, while dynamic, increases with age^8^.

In addition to age, we observed associations of mLOY with smoking, BMI, genetic ancestry, self-reported health, and a previous diagnosis of diabetes or cardiovascular disease. Smoking has been previously reported to be associated with increased mLOY risk^15,16^. The large size of the current study adds to this previous literature, clearly indicating a higher prevalence of mLOY among current smokers of all ages. The decline of mLOY prevalence with cessation suggests that the association between smoking and mLOY could be reversible and that the mechanisms contributing to mLOY are potentially modifiable. The lower overall frequency of mLOY observed in the UK Biobank in relation to our previous study of mLOY^15^ is due to the combination of a younger overall age and lower proportion of current and former smokers in the UK Biobank; a comparison of age and smoking stratified frequencies suggests no meaningful differences in frequencies of mLOY (Supplementary Fig. 2).

The association with BMI, however, is new. Potential mechanisms linking obesity to reduced frequency of mLOY are unknown and residual confounding by other factors is possible. For example, despite careful adjustment, smoking may be responsible for some of the observed association between BMI and mLOY. A recent study in the UK Biobank observed that current smokers were less likely to be obese than never smokers, and that former smokers were more likely to be obese than current smokers and never smokers^27^. Since smoking is an established risk factor for mLOY and is difficult to adjust for fully, the inverse association between mLOY and obesity may be confounded by higher frequencies of current smokers among normal weight men and higher frequencies of former smokers among obese men. The association between mLOY and ancestry has also not previously been reported; although, a previous study did report that individuals with African ancestry have lower frequencies of autosomal mosaicism^8^. It is noteworthy that 12 of the 19 previously published genetic variants associated with mLOY^17^ have higher frequencies in European populations than African populations (Supplementary Table 1)^28^ suggesting genetic differences may contribute to this observed difference in mLOY frequency. We also observed that self-reported health as well as medical conditions such as diabetes and heart attack or stroke were associated with mLOY. Past studies have observed evidence for an association of mLOY and with major cardiovascular events^20^ as well as for autosomal mosaicism with diabetes^7^, and clonal hematopoiesis with cardiovascular disease^29^; further suggesting genetic mosaicism may contribute to these diseases. An interesting point for consideration is that mLOY might not be a risk factor for these diseases, but rather these diseases might contribute to risk of mLOY; for example, age-accelerating effects of diabetes could be an important mechanism leading to higher frequencies of mLOY in men with diabetes^7^ rather than mLOY increasing risk of diabetes.

We observed that mLOY was associated with mortality in the subset of males with a higher proportion of affected cells. Variation in the proportion of affected cells across individuals with chromosomal mosaicism has been noted and discussed previously^30–32^; however, this is the first report demonstrating a relationship between the proportion of cells affected with mLOY and an outcome. We estimate that our mLRR cut points of −0.15 and −0.40 correspond to approximately 10% and 24% of cells affected with mLOY, respectively. Results using these mLRR cut points, as well as models using cubic splines, suggest that the proportion of cells with mLOY is linearly related to mortality, although the association did not reach statistical significance until a substantial proportion of cells were affected. Supporting this hypothesis, our lag analysis indicated that men with a lower proportion of cells affected by mLOY at baseline had higher risk of mortality after five years, but not earlier in follow-up, suggesting that the association may have revealed itself as the fraction of cells affected by mLOY in these men increased. These results suggest a continuum of risk for mLOY on mortality which increases as the proportion of cells affected increases. However, further support from additional studies will be needed to confirm this hypothesis.

Our analysis indicates mLOY is associated with several different risk factors for mortality and disease, including age, smoking, ancestry, and BMI. The associations, which are based on observational data, necessitate caution with regards to interpretation of the observed association between mLOY and mortality. Even with careful adjustment for age, smoking, and BMI, potential for residual confounding by these and other factors remains a possibility. Analyses that include future follow-up data from the UK Biobank in combination with parallel studies in other cohorts will be instrumental in determining whether mLOY is an independent risk factor for mortality.

Although this report and several previously published reports find notable relationships between mLOY and disease risk factors (e.g., aging and smoking) and outcomes (e.g., self-reported health, prevalent cardiovascular disease, prevalent diabetes, and all cause-mortality), little is known about the biological mechanisms leading to the formation of mLOY or the mechanisms by which mLOY may affect disease. A recent genome-wide association study suggests germline variants in cell cycle genes may alter risk of acquiring mLOY^17^, but no functional work has been performed on the identified susceptibility regions. Losing an entire chromosome in a substantial proportion of circulating blood cells likely has physiologic consequence; however, molecular studies in cells with chromosome Y loss are needed to elucidate potential biologic consequences of mLOY. It is possible that selective expansion of cellular subpopulations with mLOY may in some way alter leukocyte counts and distributions. In this way, leukocyte count may mediate certain associations between mLOY and outcomes and should not be adjusted for in analyses as a potential confounder. An altogether different possibility is that the propensity to expand sub-populations of cells with mLOY may by itself have no direct effect on disease, but is instead correlated with another age-related trait of biologic or pathophysiologic significance. For example, mLOY could be a proxy measure of stem cell population size, a number known to dwindle with age, and in this way be associated with immunity, disease risk and mortality.

In conclusion, our analysis in the UK Biobank identifies intriguing predictors of mLOY and provides insight into potential health-related consequences of postzygotic loss of the male Y chromosome. These associations merit future epidemiologic and molecular investigations targeted at understanding the impact of mosaic Y loss on men’s health.

## Electronic supplementary material

Supplementary Information

## Data Availability

All data used in this analysis is available through application to the UK Biobank.
