# Supplementary material for: Predictors of mosaic chromosome Y loss and associations with mortality in the UK Biobank
Source: Sci Rep. 2018 Aug 17;8:12316. doi: 10.1038/s41598-018-30759-1 (PMC6098142; doi:10.1038/s41598-018-30759-1)
Supplement: Supplementary file 1 — Supplementary Information [file 41598_2018_30759_MOESM1_ESM.docx]

**Supplementary Material**

**Predictors of mosaic chromosome Y loss and associations with mortality in the UK Biobank**

Erikka Loftfield, Weiyin Zhou, Barry I. Graubard, Meredith Yeager, Stephen J. Chanock, Neal D. Freedman and Mitchell J. Machiela

| **Supplementary Table 1. Allele frequencies in the 1000 Genomes European and African populations of germline genetic variants associated with mosaic loss of the Y chromosome.** | | | | | | | |
| --- | --- | --- | --- | --- | --- | --- | --- |
| **Variant** | **Coordinates (GRCh37)** | **Location** | **Nearby Gene** | **Alleles** | | **LRR Lowering Allele Frequency** | |
|  |  |  |  | **LRR Lowering** | **LRR Increasing** | **1000G Europeans** | **1000G Africans** |
| rs2736609 | chr1:156202640 | 1q22 | PMF1 | T | C | 0·351 | 0·281 |
| rs115854006 | chr3:48388170 | 3p21·31 | TREX1 | C | T | 0·964 | 0·999 |
| rs13088318 | chr3:101242751 | 3q12.3 | SENP7 | G | A | 0·348 | 0·011 |
| rs59633341 | chr3:150018880 | 3q25.1 | TSC22D2 | A | AT | 0·150 | 0·012 |
| rs56084922 | chr5:111061883 | 5q22.1 | NREP | G | A | 0·070 | 0·216 |
| rs13191948 | chr6:109634599 | 6q21 | SMPD2 | C | T | 0·571 | 0·650 |
| rs381500 | chr6:164478388 | 6q26 | QKI | C | A | 0·514 | 0·538 |
| rs4721217 | chr7:1973579 | 7p22.3 | MAD1L1 | T | C | 0·391 | 0·029 |
| rs35091702 | chr8:30279470 | 8p12 | RBPMS | C | CAAAAAAG | 0·775 | 0·886 |
| rs4754301 | chr11:108048541 | 11q22.3 | NPAT | A | G | 0·621 | 0·334 |
| rs10687116 | chr13:41678081 | 13q14.11 | WBP4 | AGATG | A | 0·843 | 0·899 |
| rs1122138 | chr14:96180242 | 14q32.13 | TCL1A | C | A | 0·827 | 0·626 |
| rs137952017 | chr14:101176090 | 14q32.2 | DLK1 | C | CT | 0·831 | 0·826 |
| rs12448368 | chr16:81044947 | 16q23.2 | CENPN | C | T | 0·125 | 0·062 |
| rs78378222 | chr17:7571752 | 17p13.1 | TP53 | G | T | 0·013 | 0·000 |
| rs77522818 | chr17:47817373 | 17q21·33 | FAM117A | A | T | 0·939 | 0·928 |
| rs11082396 | chr18:42080720 | 18q12.3 | SETBP1 | C | T | 0·146 | 0·078 |
| rs17758695 | chr18:60920854 | 18q21·33 | BCL2 | C | T | 0·970 | 0·999 |
| rs60084722 | chr20:30355738 | 20q11·21 | TPX2 | CT | C | 0·781 | 0·626 |

Abbreviations: GRCh37, Genome Reference Consortium Human Build 37; LRR, log_2_ intensity ratio of the Y chromosome; 1000G, 1000 Genomes

| **Supplementary Table 2. Association of mosaic loss of the Y chromosome with all-cause mortality excluding key population subgroups** | | | | | | | | | | | |
| --- | --- | --- | --- | --- | --- | --- | --- | --- | --- | --- | --- |
|  | mLOY (mLRR < -0·15) | | |  | mLOY (mLRR < -0·40) | | |  | mLRR ^d^ | | |
|  | HR ^a^ | 95% CI | P-value |  | HR ^a^ | 95% CI | P-value |  | HR ^a^ | 95% CI | P-value |
| Excluding mLRR > +0·15 ^b^ | 1·11 | (1·00-1·24) | 0·06 |  | 1·40 | (1·11-1·76) | 0·004 |  | 1·02 | (1·00-1·04) | 0·01 |
| Excluding prevalent chronic disease ^c^ | 1·06 | (0·91-1·22) | 0·47 |  | 1·49 | (1·11-2.00) | 0·008 |  | 1·03 | (1·01-1·05) | 0·008 |
| ^a^ Multivariable model is adjusted for age (as the underlying time metric), detailed smoking history (25-level variable incorporating current smoking status, smoking intensity (current and former smokers); time since quitting (former smokers), and cigar and pipe use (current and former smokers)); time to first cigarette among current smokers (<5 minutes, 5 to 15 minutes, 30 minutes to 1 hour, or >1 hour)); race/ethnicity (white, black, Asian, mixed, or other race); alcohol drinking (never drinker, former drinker, infrequent drinker (<1 drink/week), occasional drinker (>1 drink/week but <1 drink/day), moderate daily drinker (1 to 3 drinks/day), or heavy daily drinker (>3 drinks/day); general health status (excellent, good, fair, or poor); education level (college or university degree, A levels/AS levels or equivalent, O levels/GCSEs or equivalent, CSEs or equivalent, NVQ or HND or HNC equivalent, or other professional qualifications); body mass index (<18·5, 18·5 to <25, 25 to <30, 30 to <35, or ≥35 kg/m^2^); and physical activity (>10 minutes of moderate of vigorous activity 0, 1-2, 3-4, or ≥5 days/week) | | | | | | | | | | | |
| ^b^ Excluding individuals with a Y chromosome gain event (mLRR > +0·15, n=205) | | | | | | | | | | | |
| ^c^ Excluding individuals with a self-reported diagnosis of diabetes, cancer (other than non-melanoma skin cancer), heart attack or stroke or missing data on self-reported history (n=36,382) | | | | | | | | | | | |
| ^d^ Scaled by the - (standard deviation) of mLRR such that the HR corresponds to a one standard deviation decrease in mLRR | | | | | | | | | | | |
| Abbreviations: CI, confidence interval; HR, hazard ratio; mLOY, mosaic loss of the Y chromosome; mLRR, median log_2_ intensity ratio of the Y chromosome | | | | | | | | | | | |

| **Supplementary Table 3. Lag analysis of the association of mosaic loss of the Y chromosome with all-cause mortality for more than 5 years of follow-up** | | | | | | | | | | | |
| --- | --- | --- | --- | --- | --- | --- | --- | --- | --- | --- | --- |
|  | mLOY (mLRR < -0·15) | | |  | mLOY (mLRR < -0·40) | | |  | mLRR ^b^ | | |
| Years of follow-up | HR ^a^ | 95% CI | P-value |  | HR ^a^ | 95% CI | P-value |  | HR ^a^ | 95% CI | P-value |
| 0 to 5 years (n=223,338) | 1·00 | (0·86-1·16) | 0·98 |  | 1·37 | (1·03-1·83) | 0·03 |  | 1·01 | (0·99-1·03) | 0·36 |
| >5 to 10 years (n=218,238) | 1·26 | (1·06-1·49) | 0·008 |  | 1·35 | (0·92-1·97) | 0·12 |  | 1·03 | (1·00-1·06) | 0·03 |
| ^a^ Multivariable model is adjusted for age (as the underlying time metric), detailed smoking history (25-level variable incorporating current smoking status, smoking intensity (current and former smokers); time since quitting (former smokers), and cigar and pipe use (current and former smokers)); time to first cigarette among current smokers (<5 minutes, 5 to 15 minutes, 30 minutes to 1 hour, or >1 hour)); race/ethnicity (white, black, Asian, mixed, or other race); alcohol drinking (never drinker, former drinker, infrequent drinker (<1 drink/week), occasional drinker (>1 drink/week but <1 drink/day), moderate daily drinker (1 to 3 drinks/day), or heavy daily drinker (>3 drinks/day); general health status (excellent, good, fair, or poor); education level (college or university degree, A levels/AS levels or equivalent, O levels/GCSEs or equivalent, CSEs or equivalent, NVQ or HND or HNC equivalent, or other professional qualifications); body mass index (<18·5, 18·5 to <25, 25 to <30, 30 to <35, or ≥35 kg/m^2^); and physical activity (>10 minutes of moderate of vigorous activity 0, 1-2, 3-4, or ≥5 days/week) | | | | | | | | | | | |
| ^b^ Scaled by the - (standard deviation) of mLRR such that the HR corresponds to a one standard deviation decrease in mLRR | | | | | | | | | | | |
| Abbreviations: CI, confidence interval; HR, hazard ratio; mLOY, mosaic loss of the Y chromosome; mLRR, median log_2_ intensity ratio of the Y chromosome | | | | | | | | | | | |

**Supplementary Figure 1. Plot of median log_2_ intensity ratio of the Y chromosome (mLRR) values for all UK Biobank men in our analytical set by age of blood draw. Solid line represents expected baseline mLRR of 0 and dashed lines represent mLRR cutoff values of +0·15, -0·15 and -0·40, respectively.**


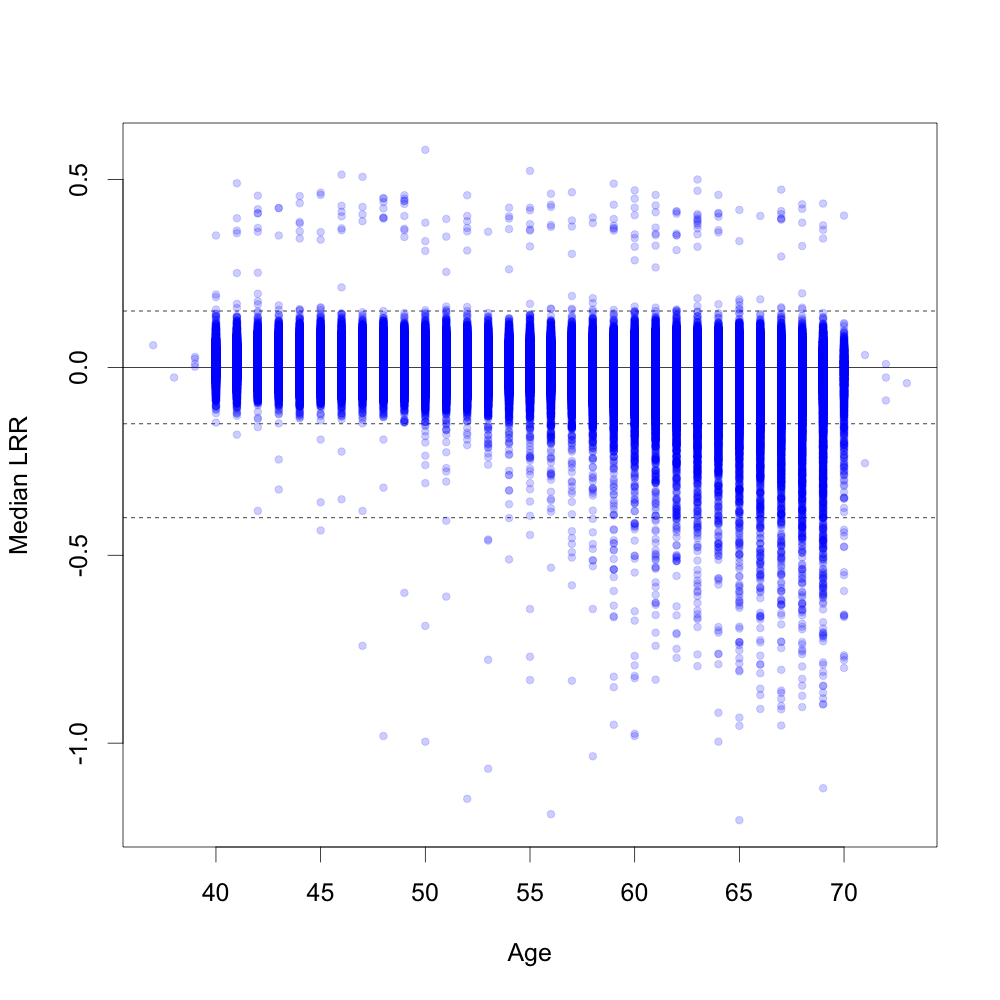


**Supplementary Figure 2. Comparison of the frequency of mLOY in the UK Biobank to a previous study by Zhou et al^15^ by 5-year age group and smoking status. Bar height represents mLOY frequency estimates and plotted whiskers represent 95% confidence intervals.**
